# Brain state dependent stimulus information in the auditory thalamocortical system

**DOI:** 10.1186/1471-2202-16-S1-P99

**Published:** 2015-12-18

**Authors:** Jon Bamber, Shuzo Sakata, J Michael Herrmann

**Affiliations:** 1 Institute for Adaptive and Neural Computation, University of Edinburgh, Edinburgh, EH8 9AB, UK; 2 Strathclyde Institute of Pharmacy and Biomedical Sciences, University of Strathclyde, Glasgow, G4 0RE, UK; 3 Institute of Perception, Action and Behaviour, University of Edinburgh, Edinburgh, EH8 9AB, UK

## 

Activity in the absence of stimuli is ubiquitous across the thalamocortical system (TS), with patterns of spontaneous activity reflecting ongoing behavioural state. Under anaesthesia and during deep sleep the TS operates in an inactivated state (characterised by low frequency high amplitude oscillations in local field potential (LFP)) in which neurons collectively alternate between periods of local silence and high synaptic activity. During wakefulness and REM sleep, however, the TS operates in an activated state (characterised by high frequency low amplitude oscillations in LFP) in which neurons fire in a sustained desynchronised manner. Such brain states may be indicative of different modes of neural processing, but the effect of brain state on neural processing remains unclear.

Here we analyse data recorded in the auditory TS of urethane anaesthetised rats subjected to single-click acoustic stimulation over a range of intensities, presented in both the inactivated state (natural under the anaesthesia) and the activated state (induced through electrical stimulation of the basal forebrain). Evoked spike trains were identified for single units in the auditory thalamus and across depths of the primary auditory cortex. Mutual information (MI) between stimulus and response was then computed for spike probability, counts and timing.

Evoked spiking activity was observed to last around 300ms, consisting of distinct initial and secondary (*rebound*) activity. Analysis of responses of single units over a full 300ms window showed that spike count and timing measures gave little more MI than response probability, suggesting that stimulus intensity is primarily encoded probabilistically, at least at the level of the single unit. Moreover, whilst many single units are uninformative of stimulus intensity, the most informative thalamorecipient (TC) and infragranular (IF) single units show increased MI in the activated state. Additionally, upon temporally partitioning data according to initial or rebound activity, we see that TC units are more informative in the activated state during initial activity whereas IF units are more informative in the activated state during rebound activity, suggesting that information loss in the inactivated state may be cumulative in time as sensory signals propagate through neural circuits.

